# Antioxidant, Anti-Inflammatory, Antagonistic, and Probiotic Properties of Lactic Acid Bacteria Isolated from Traditional Algerian Fermented Wheat

**DOI:** 10.3390/microorganisms13081852

**Published:** 2025-08-08

**Authors:** Rachida Benguiar, Rachida Benaraba, Chayma Farhat, Habib Chouchane, Djilali Boughaddou, Fethi Belalem, Ameur Cherif

**Affiliations:** 1 Department of Nutrition and Agri-food Technology, Faculty of Nature and Life Sciences, University of Tiaret, Tiaret 14000, Algeria; 2 Laboratory of Improvement and Valorization of Local Animal Productions, Institute of Veterinary Sciences, University of Tiaret, Tiaret 14000, Algeria; rachida.benaraba@univ-tiaret.dz; 3 Department of Biology, Faculty of Nature and Life Sciences, University of Tiaret, Tiaret 14000, Algeria; jilali.boughaddou@gmail.com (D.B.); belalemfethi5@gmail.com (F.B.); 4 University of Manouba, ISBST, BVBGR-LR11ES31, Biotechpole Sidi Thabet, Ariana 2020, Tunisia; chaymafarhat027@gmail.com (C.F.); habib.chouchane@isbst.uma.tn (H.C.); cherifameur@gmail.com (A.C.)

**Keywords:** lactic acid bacteria, probiotic, traditional fermented wheat, antioxidant activity, antibacterial activity, anti-inflammatory activity

## Abstract

This study focuses on the identification of three lactic acid bacteria isolates obtained from traditional Algerian fermented wheat as well as the evaluation of their biological activities, mainly their probiotic, antimicrobial, anti-inflammatory, and antioxidant properties. These isolates were identified through phenotypic and genotypic characterizations. It was found that isolate LB3 was *Lactiplantibacillus plantarum*, while isolates LB1 and LB2 were identified as *Weissella confusa*. It was observed that the strains LB1, LB2, and LB3 are capable of maintaining their growth at pH 3.0 and in bile salts after 4 h, with individual survival rates ranging from 41% to 90% depending on the strain. Furthermore, their co-aggregation capacity with *Staphylococcus aureus* ATCC6528 indicated a percentage higher than 50%. The three strains displayed powerful inhibitory effects against pathogenic bacteria, showing inhibition rates of 5% to 40%. They also exhibited significant anti-inflammatory activity ranging from 20% to 39%. All three lactic acid bacteria (LAB) isolates exhibited significant antioxidant activity. Their intact cells demonstrated a high ability to scavenge DPPH radicals and possessed substantial ferric reducing power, while their intracellular extracts showed high levels of glutathione (GSH). Additionally, they exerted a protective effect against plasma lipid peroxidation, with inhibition rates ranging from 20% to 39%. These findings suggest that these strains possess promising probiotic potential as future therapeutic agents to be used in the development of novel functional fermented foods.

## 1. Introduction

The gut microbiota is crucial in maintaining an organism’s homeostasis. An imbalance in the latter induces an alteration in the functioning of the immune system, promoting the development of certain pathologies, such as allergies, chronic inflammatory diseases, and intestinal infections [1,2]. The causes of this dysbiosis are complex and probably attributable to several factors, such as the environment, genetics, and diet. Indeed, its modulation through diet can be a key factor in ensuring and restoring its balance and consequently maintaining the host’s health and well-being. For this reason, researchers are very interested in biological products such as beneficial bacteria, primarily probiotics. These are living microorganisms that, when administered in adequate quantities, would have a beneficial effect on the host’s health [3].

Probiotics are often praised for their beneficial effects on health as well as their ability to influence diseases such as cancer and obesity, which are associated with imbalances in the gut flora [4]. Their effectiveness is mainly linked to the selection of the species and even the specific bacterial strain. According to Yadav and Shukla [5], lactic acid bacteria (LAB) stand out among the selected live bacterial strains for their crucial role in maintaining intestinal balance [6]. Due to their numerous health benefits, lactic acid bacteria are considered promising probiotic candidates. However, it is necessary to emphasize that not all bacteria can be considered probiotics, and it is essential to examine their probiotic properties as well as their safety. These microbes should have considerable resistance to the stomach’s highly acidic conditions in order to survive [7]. Furthermore, their ability to adhere to the gastrointestinal tract (intestinal mucosa and epithelial cells) prevents their elimination by intestinal motility. This adhesion allows them to reproduce, colonize, and affect the immune system while also competitively eliminating pathogens [8].

This ability demonstrates their aptitude for food bioconservation and their potential to be used as a starter culture in the fermentation process under controlled conditions. During fermentation, LAB synthesize a number of compounds, such as exopolysaccharides, aromatic compounds, organic acids, etc., which extend the shelf life of food and improve its sanitary, sensory, and nutritional properties, in addition to increasing the antioxidant capacity of the fermented food. This increase is mainly due to the depolymerization of phenolic compounds [9]. Therefore, the valorization and investigation of the probiotic potential of LAB isolated from various local fermented foods and products have attracted scientific interest in recent years. Fermented wheat, which is one of the food substrates stimulating their development, appears to be a promising resource. It provides natural support for the separation and analysis of LAB.

In Algeria, Hamoum, a traditionally fermented wheat, is naturally produced in an underground silo known as a Matmora [10]. In addition to its sensory and gustatory attributes, fermentation enhances the nutritional properties of wheat and stimulates the multiplication of beneficial microorganisms. According to Nithya et al. [11], fermented wheat is recognized for its preventive virtues against intestinal pathophysiological dysfunctions. Lactic bacteria derived from fermented wheat could represent a promising solution commonly used in the medical and food sectors. In this context, the present study focuses on the identification of three selected LAB isolates obtained from traditional Algerian fermented wheat, as well as the in vitro evaluation of their probiotic, antibacterial, anti-inflammatory, and antioxidant properties.

## 2. Materials and Methods

### 2.1. Lactic Acid Bacteria Isolates

Three bacterial isolates were obtained from traditional fermented wheat, harvested in Rahwia in the Tiaret region of Algeria. The pure culture isolates, previously maintained in 30% (*v*/*v*) of glycerol and stored at −20 °C, were transferred into deMan, Rogosa, Sharpe (MRS) (Parodonsia, Conda S A, Pamplona, Spain) broth and incubated at 37 °C for 24 h, either under aerobic conditions (LB1, LB2) or under anaerobic conditions (LB3).

### 2.2. Indicator Microorganisms for Antimicrobial Activity Assay

The strains used for the evaluation of antibacterial activity are *Escherichia coli* ATCC 10536, *Bacillus subtilis* ATCC 6633, and *Bacillus cereus* ATCC 10876, and *Staphylococcus aureus* ATCC6528 provided by SAIDAL and the Mostapha Bacha Hospital in Algiers, as well as an isolate of *Pseudomonas aeruginosa* from a canine infection at the Veterinary Institute of Tiaret.

### 2.3. Phenotypic Identification of the Isolates

Colony morphology of the three strains was examined on MRS agar following 24 h of incubation at 37 °C under aerobic or anaerobic conditions. Gram staining was also performed [12]. The catalase test and the fermentation type were evaluated according to the method described by Delarass [13]. Proliferation at various temperatures was recorded in MRS broth following incubation at 10 °C, 30 °C, and 45 °C for 72 h [14]. The resistance of bacterial isolates to NaCl was assessed in MRS broth with 6.5% and 9.5% NaCl at 30 °C for 48 h [15].

### 2.4. Molecular Identification of Bacteria Using 16S rRNA Gene Sequencing

The genomic DNA of LAB isolates from traditional Algerian fermented wheat was extracted according to the optimized protocol at the Laboratory of Biotechnology and Bio-Geo-Resource Valorization, Higher Institute of Biotechnology Sidi Thabet, Tunis, using the phenol-chloroform technique. The amplification and sequencing of the 16S rRNA gene were carried out utilizing universal primers (27f 5′AGAGTTTGATCMTGGCTCAG 3′ and 1492R 5′ GGTTACCTTGTTACGACTT3′). In this process, 1 µL of extracted DNA was diluted to 1/10 and added to the reaction mixture containing 2.5 µL of PCR buffer (10×), 0.2 µL of deoxyribonucleoside triphosphates, 0.3 µL of each primer, and 0.2 µL of Taq DNA polymerase, then adjusted to 25 µL with distilled water. PCR reactions were performed on a thermocycler (T100 BIO RAD, Hercules, CA, USA) using the following program: an initial denaturation cycle at 95 °C for 3 min, followed by 35 cycles of 30 s denaturation at 95 °C, 1 min of hybridization at 57.5 °C, 1 min of elongation at 72 °C, and a final elongation cycle at 72 °C for 10 min.

Sequencing was performed using the automated Sanger technique. The sequence obtained from the gene coding for 16S rRNA was compared with the homologous sequences of reference microbial species listed in the GenBank, using NCBI BLAST^+^2.16.0 (http://blast.ncbi.nlm.nih.gov accessed on 23 May 2025). An evolutionary tree was also developed using MEGA 11 software to identify the most similar bacterial species using the neighbor-joining method [16]. The nucleotide sequences have been added to the GenBank with accession numbers PV652951, PV653095, and PV653185 for the isolated bacteria LB1, LB2, and LB3, respectively.

### 2.5. Probiotic Properties Assessment

#### 2.5.1. Tolerance to Acidity

The tolerance of the strains to acidity was assessed according to Anandharaj et al. [17] MRS medium was adjusted to pH 3.0 and pH 6.5, inoculated with overnight cultures, and incubated at 37 °C. The growth was established over 3 h. The survival rate (%) was determined using the pour plate count method on MRS agar after incubation for 0 and 3 h.

#### 2.5.2. Bile Salts Tolerance

The bile salt resistance of the strains was assessed according to the method described by Anandharaj et al. [17]. The MRS medium supplemented with 0.3% (*w*/*v*) bile salts was inoculated with an 18 h culture of bacterial isolates (10^8^ CFU/mL) and the incubation was carried out at 37 °C. The viability rate (%) was assessed utilizing the colony counting technique on MRS agar after incubation for 0 and 4 h.

#### 2.5.3. Antibiotic Sensitivity

The antibiotic sensitivity of strains was determined according to Tarique et al. [18]. The bacterial suspensions of each isolate adjusted to 10^6^ CFU/mL were introduced and kept at 37 °C for incubation after the placement of antibiotic disks. The inhibition diameters were evaluated following the Clinical and Laboratory Standards (CLSI) guidelines [19].

#### 2.5.4. Auto-Aggregation

The surface cell auto-aggregation was assessed according to the modified protocol of Abdulla et al. [20]. The overnight cultures of bacterial isolates were subjected to centrifugation for 15 min at 4500× *g* (4 °C) and then were rinsed with phosphate-buffered saline (PBS) (pH 7.2) and adjusted to 10^8^ CFU/mL (A_0_). Then, 4 mL of the cell suspension was mixed, maintained at 37 °C for 3 h incubation, and the absorbance of the supernatant was determined at 625 nm (A_1_). The percentage of bacterial cell auto-aggregation was measured using Equation (1):

A% = 1 − (A_1_/A_0_) × 100 (1)

A_1_ is the absorbance at 3 h and A_0_ is the absorbance at 0 h.

#### 2.5.5. Co-Aggregation

The co-aggregation rates between the isolated strains and two pathogenic bacteria (*S. aureus* and *E. coli*) were assessed according to the protocol of Abushelaibi et al. [21]. The same volumes of the lactic and pathogenic strain suspensions were mixed, vortexed, and then subjected to 3 h of incubation at 37 °C. The absorbance of the selected LAB strains (Ax), the pathogenic strains (Ay), and the mixture [A(x+y)] was recorded at 620 nm. The rate of co-aggregation was measured according to Formula (2):

%= {(A_X0_ + A_Y0_)/2 − A(x+y)/(A_X0_ + A_Y0_)/2} × 100, (2)

where A_X0_ is the absorbance in the presence of LAB strains at 0 h, A_Y0_ is the absorbance in the presence of pathogenic bacteria at 0 h, and A(x+y) is the absorbance of the mixture after 3 h of incubation.

#### 2.5.6. Hemolytic Activity

The bacterial cultures were streaked on the surface of horse blood agar (5% *v*/*v*) and incubated for 48 h at 37 °C. The degradation of the blood was noted by the formation of distinct halos (β hemolysis) or greenish areas (α hemolysis) surrounding the colonies [22].

#### 2.5.7. Proteolytic Activity

The proteolytic properties of the strains were assessed using the disk technique on MRS agar containing 10% skim milk. Disks impregnated with 15 μL of each bacterial culture were placed on the agar and then incubated at 30 °C and 37 °C for 24 h. The diameters of the hydrolysis zones were measured to classify proteolytic activity as follows: very high for a halo greater than 10 mm, moderate for a halo of 3 to 10 mm, and low for a halo less than 3 mm [23].

### 2.6. Determination of Antibacterial Activity Using Microdilution Method

The culture supernatant of bacterial isolates in MRS broth was recovered after centrifugation at 4000 rpm for 10 min. This supernatant was then diluted to different concentrations in Mueller–Hinton broth, and 100 µL of each generated concentration was deposited into the wells of a microplate. Then, each well was inoculated with 10^6^ CFU/mL of a suspension of pathogenic bacteria. The wells in the first row of the same microplate received Mueller–Hinton medium inoculated with the same concentration of bacteria and without supernatant. The prepared microplates underwent 21 h of incubation at 37 °C. After incubation, 20 μL of 2,3,5 triphenyltetrazolium (Sigma-Aldrich, St. Louis, MO, USA) was added to observe the bacterial proliferation [24]. The inhibitory activity of the bacterial isolates was expressed as an inhibition rate (IR%).

### 2.7. Assessment of Anti-Inflammatory Properties

The anti-inflammatory properties of the LAB isolates were assessed according to Kar et al. [25]. Overnight cultures of each bacterial isolate were prepared. After centrifugation at 6000 rpm for 10 min at 4 °C, the bacterial cell pellets were subjected to three successive washes with 500 μL of sterile PBS (20 mM, pH = 7.4). These were then reconstituted in 500 μL of sterile PBS. The total number of cells was adjusted to 10^9^ CFU/mL (OD ≈ 1.2) [26]. The assessment of the capacity to inhibit protein denaturation was carried out by preparing three solutions: Solution 1, composed of 450 μL of BSA (at 5% *v*/*v*) and 50 μL of bacterial suspension; Solution 2, composed of 450 μL of BSA and 50 μL of bidistilled water as a control; and Solution 3, composed of 450 μL of BSA and 50 μL of sodium diclofenac (100 mg/mL). After adjusting each solution to pH 6.3, they were incubated for 20 min at 37 °C and then subjected to 75 °C for 3 min. The absorbance was determined after the cooling process, and Formula (3) was used to evaluate the rate of protein denaturation inhibition,

I% = 100 × [1 − (A_2_/A_1_)], (3)

where A_1_ is the absorbance of the test solution and A_2_ is the absorbance of the positive control.

### 2.8. Antioxidant Activity

#### 2.8.1. Evaluation of Ferric Reducing Antioxidant Power (FRAP)

The test for the evaluation of ferric-reducing antioxidant power (FRAP) was developed by Benzie and Strain [27] and was based on the reduction of iron under acidic conditions (pH 3.6) in a solution containing acetate buffer, 2,4,6-Tris(2-pyridyl)-s-triazine (TPTZ), and FeCl_3_(Sigma-Aldrich, St. Louis, MO, USA). This reaction forms a blue TPTZ-Fe++ complex, the color intensity of which was measured. The FRAP solution was prepared from acetate buffer at pH 3.6, TPTZ at 10 mM, and FeCl_3_ at 20 mM, and was incubated at 37 °C. After that, a volume of 100 μL of each solution was adjusted to 900 µL of the FRAP solution and kept at room temperature for 30 min of incubation. The concentration of ferric-reducing power for each bacterial suspension was calculated using a calibration curve performed with FeSO_4_.

#### 2.8.2. Evaluation of 2,2-Diphenyl-1-Picrylhydrazyl (DPPH•) Scavenging

The evaluation of 2,2-diphenyl-1-picrylhydrazyl (DPPH•) (Sigma-Aldrich, St. Louis, MO, USA) scavenging was performed according to the protocol described by Sanchez-Moreno et al. [28]. A volume of 750 μL of each bacterial suspension was added to 750 μL of the methanolic DPPH• solution at 4 mg/mL. The reaction mixture was incubated at room temperature and in the dark for 50 min, and then the absorbance was measured at 517 nm. The ability of the bacterial isolates to scavenge DPPH• was expressed as a percentage of inhibition, calculated using Equation (4):

% inhibition of the DPPH• radical = [(A_1_ − A_2_)/A_1_] × 100, (4)

where A_1_ is the absorbance of the control and A_2_ is the absorbance of the sample.

#### 2.8.3. Lipid Peroxidation Evaluation

The evaluation of lipid peroxidation was performed according to the protocol of Bekkouche et al. [29]. In this process, 160 μL of each bacterial suspension was added to a reaction mixture consisting of 40 μL of a copper sulfate solution (CuSO_4_ at 0.33 mg/mL) and 160 μL of human plasma. The mixture was incubated at 50 °C for 12 h. Two controls were used: a negative control (160 μL of plasma + 160 μL of distilled water) and a positive control (160 μL of plasma + 160 μL of distilled water + 40 μL of CuSO_4_). After 12 h of incubation, 200 mL of the reaction solution was added to 800 μL of a thiobarbituric acid mixture (0.375% (*w*/*v*)), trichloroacetic acid (20%), 2,6-di-tert-butyl-4-methylphenol (0.01%)(Sigma-Aldrich, St. Louis, MO, USA), and HCl (1N) (20%) and subjected to 15 min of incubation at 100 °C. After cooling, 2 mL of butanol-1 was used to extract the complex that had formed. After centrifugation at 4000 rpm for 10 min, the absorbance was read at 532 nm. Malondialdehyde (MDA) concentration was determined utilizing the 1,3,3-tetraethoxypropane curve, and the MDA inhibition rate was evaluated using Equation (5):

MDA (%) = [(A_0_ − A_1_)/A_0_] × 100, (5)

where A_0_ is the MDA concentration of the positive control and A_1_ is the MDA concentration of the sample.

#### 2.8.4. Determination of Reduced Glutathione (GSH) Levels

##### Preparation of Intracellular Extracts of LAB Isolates

Based on Su et al. [26], overnight cultures of each bacterial isolate were centrifuged at 6000 rpm for 10 min at 4 °C, and the cells were rinsed three times with sterile PBS, then adjusted to 10^8^ CFU/mL. Cell suspensions were subjected to ultrasonic disruption. Sonication was conducted for five intervals of one minute each in an ice bath. Cell debris was eliminated using centrifugation at 10,000 rpm for 20 min at 4 °C, and the resultant intracellular extracts were utilized to quantify total proteins and GSH.

##### GSH Assay

In the GSH assay, 500 μL of intracellular extract from bacterial isolates was mixed with 750 μL of PBS (0.05N, pH 8.0) and 250 μL of Ellman’s reagent. The mixture was kept for 15 min at room temperature. The optical density reading was performed at 412 nm. The concentration of GSH was determined using a calibration curve of reduced GSH (Sigma-Aldrich, St. Louis, MO, USA).

### 2.9. Statistical Analysis

Data analysis was conducted using Statistica software (version 8, Statsoft, Tulsa, OK, USA). One-way analysis of variance (ANOVA) was used to perform the comparison of means. This analysis was followed by Tukey’s post hoc test to determine significant differences and compare the means. The differences were considered statistically significant for a *p*-value less than 0.05. All experiments were performed in triplicate.

## 3. Results

### 3.1. Identification of Bacterial Isolates

#### 3.1.1. Morphological, Biochemical, and Physiological Characteristics

The LAB isolates exhibited morphological diversity. The colonies of isolates LB1 and LB2 were small, round, smooth, and white, whereas that of LB3 was medium-sized, smooth, and white. At the microscopic examination, the three strains were Gram-positive: LB1 and LB2 were cocci (isolated or in pairs), while LB3 was rod-shaped (isolated or in clusters). The results regarding the biochemical characteristics indicated that these bacteria were catalase-negative. The strains LB1 and LB2 hydrolyzed arginine (ADH+), unlike the strain LB3, which did not hydrolyze arginine. Regarding fermentation, LB1 and LB2 were heterofermentative, while LB3 was homofermentative. Physiological identification revealed that the three strains showed growth at the tested temperature, and they were all capable of growing in NaCl concentrations of 6.5% (Table 1).

#### 3.1.2. Molecular Identification

**Figure 1:**
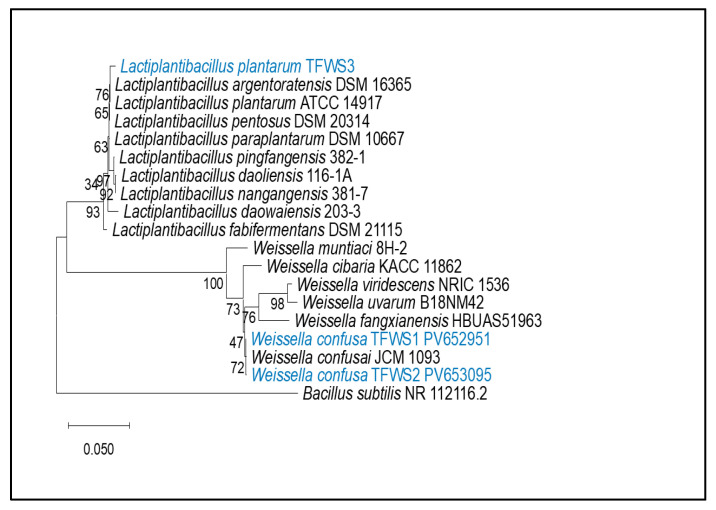
Phylogenetic tree illustrating the relative positions of lactic acid bacteria obtained from traditional Algerian fermented wheat. *Bacillus subtilis* served as an outgroup.

The sequence similarity results revealed a 99% homology with the sequences of *Weissella confusa* (isolates LB1 and LB2), while isolate LB3 belongs to *Lactiplantibacillus plantarum* isolate LB3). The phylogenetic tree is shown in Figure 1.

### 3.2. Tolerance to Acidic pH and Bile Salts

The results revealed that the isolates LB1 and LB3 exhibited better tolerance, with high survival rates estimated at 54% and 52.93 ± 0.003%, respectively, at pH 3.0 for 3 h, compared to strain LB2 and normal conditions (pH 6.5). Furthermore, the results regarding bile salt resistance (0.3%) demonstrated that all strains obtained from fermented wheat revealed a high ability to persist for 4 h in the presence of 0.3% bile salts, indicating survival rates of 89%, 76%, and 98%, respectively (Table 2).

### 3.3. Antibiotic Sensitivity

The findings indicated that the LAB isolates resisted metronidazole, ceftazidime, and streptomycin, with an absence of inhibition zones. On the other hand, they did not show resistance to the antibiotic chloramphenicol, with inhibition diameters greater than 15 mm (Table 3).

### 3.4. Co-Aggregation and Auto-Aggregation

The findings revealed that all bacterial strains exhibited a strong auto-aggregation capacity, with rates varying between 64.5 ± 0.76% and 73.83 ± 0.33%. However, the co-aggregation results mentioned in Table 4 indicate that strain LB3 exhibited a high co-aggregation potential with *E. coli*, evaluated at 27.85 ± 0.41%, while strains LB1 and LB2 had lower co-aggregation ability, at 6.78 ± 0.16% and 6.19 ± 0.6%, respectively. As for co-aggregation with *S. aureus*, all selected strains, LB1, LB2, and LB3, demonstrated a considerable capacity ranging from 50.2 ± 0.55 to 60 ± 1.2%.

### 3.5. Hemolytic Activity

The results regarding the hemolytic activity indicated that no hemolytic activity was detected. No zone of hemolysis was observed around the colonies, indicating that all the isolates are characterized by a γ-hemolysis type.

### 3.6. Proteolytic Activity

The results indicated that all of the strains showed strong proteolytic activity on MRS agar supplemented with 10% skim milk at a temperature of 30 and 37 °C, with values ranging between 19.3 ± 0.3 mm and 23.5 ± 1.42 mm (Table 5).

### 3.7. Antibacterial Activity

**Figure 2:**
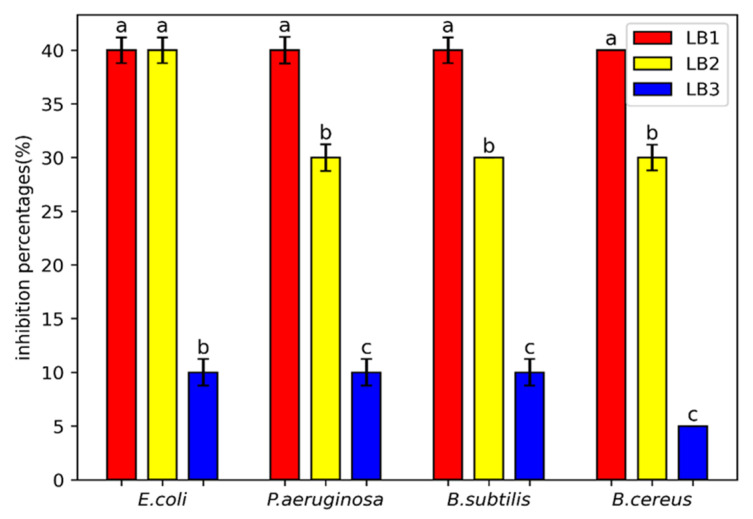
Inhibition rate of bacterial isolates against pathogenic bacteria. The results are expressed as means ± SE (*n* = 3); letters indicate significantly different between strains (*p* < 0.05).

The results of the inhibition rate evaluation by microdilution showed that each strain had a different spectrum of antibacterial activity. The supernatant of the LB3 strain revealed the inhibitory effect against *B. cereus* (IR of 5%), followed by *B. subtilis*, *E. coli*, and *P. aeruginosa* (IR of 10%). In contrast, the supernatants of isolates LB1 and LB2 displayed an IR between 30 and 40% (Figure 2).

### 3.8. Anti-Inflammatory Activity

**Figure 3:**
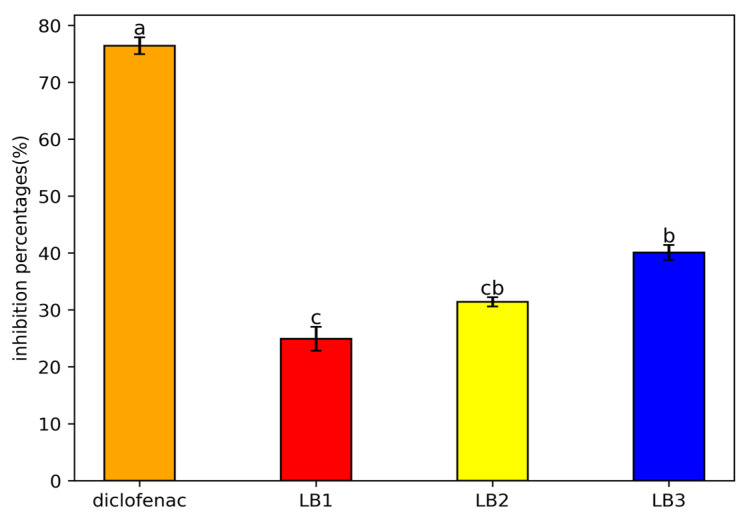
Anti-inflammatory activity of the bacterial isolates. The results are expressed as means ± SE (*n* = 3); letters indicate significantly different between strains (*p* < 0.05).

The results revealed that strain LB3 was the most active, with an inhibition rate of 40%, while the LB1 and LB2 isolates showed inhibitions of 25% and 33%, respectively. However, this capacity remains significantly lower than that observed with the diclofenac standard (Figure 3).

### 3.9. Antioxidant Activity

The results indicated that isolate LB3 exhibited antioxidant activity, reflected by greater and significant ferric-reducing power with a concentration of 311.99 ± 1.55 μmol/L. This value is higher than those observed for the other bacterial isolates, which ranged between 146.97 ± 1.25 and 285.18 ± 1.58 μmol/L (Table 6). However, the three strains had high DPPH^•^ radical-scavenging rates, ranging from 80.58 ± 0.15% to 86.75 ± 0.65%. The concentrations of GSH in the three intracellular extracts were very high and similar, with rates of 1.02 ± 0.03, 1.08 ± 0.05, and 1.12 ± 0.04 μmol/mg of protein, respectively.

The three bacterial strains (LB1, LB2, and LB3) possessed a protective antioxidant capacity against the plasma lipid peroxidation, with values ranging between 27.83 ± 0.0025% and 39 ± 0.002%. We observed that strain LB1 was the most active strain, with a percentage of around 39.18 ± 0.002%, almost identical to that of strain LB3 (Table 6).

## 4. Discussion

This study aims to identify the phenotypic and genotypic characteristics of certain lactic acid bacteria isolated from traditional Algerian fermented wheat and to investigate their probiotic and biological properties. The results obtained regarding the phenotypic characteristics of the bacterial isolates coded as LB1 and LB2 confirm the phenotypic identification characteristics of *Weissella*, and for the isolate coded as LB3, they confirm that it belongs to the genus *Lactiplantibacillus*. These are identical to the main identification characteristics presented by Kandler and Weiss [30]. Moreover, the results of 16S rRNA gene sequencing confirmed that these bacteria belong to these two genera, namely, *Weissella* and *Lactiplantibacillus*. The genera we identified in our samples corroborate those of Tahlaïti et al. [31], as well as Chadli et al. [32], who studied the molecular identification of LAB derived from Algerian Hamoum. These authors found that most of the isolates belong to *Leuconostoc* sp., *Pediococcus* sp., *Lactobacillus* sp., and *Enterococcus* sp.

The resistance of bacterial isolates to an acidic pH can be attributed to their ability to produce acids and other metabolites that stabilize their environment. This tolerance is reinforced by internal pH regulation mechanisms, such as proton exchange, allowing lactobacilli to survive in an acidic environment [33]. These findings are consistent with previous studies which demonstrated that *Lactiplantibacillus plantarum*, *Weissella confusa*, and *Weissella cibaria* have high survival rates in acidic environments [31,34]. We also noticed the tolerance of these bacteria to bile salts (0.3%). These results corroborate those obtained by Angelescu et al. [35] and Chadli et al. [32], who observed strong survival rates of LAB in the presence of bile salts.

The high auto-aggregation of the three strains can be attributed to several factors, including the production of polysaccharides, electrostatic interactions between surface charges, as well as Van der Waals forces and hydrophobic bonds [36]. Anandharaj et al. [17] reported that *Lactobacillus* sp. and *Weissella* sp. exhibit auto-aggregation rates between 18% and 79%. Similarly, the significant co-aggregation capacity between probiotic bacteria and Gram-positive pathogens is explained by their morphological similarity, particularly the presence of peptidoglycan and their hydrophobic nature [37]. This co-aggregation allows probiotics to release antimicrobial agents near pathogens, creating a hostile environment that inhibits their growth [33]. Furthermore, the results of the proteolytic activity corroborate those obtained by N’tcha et al. [38], who revealed that *Limosilactobacillus fermentum*, *Lacticaseibacillus casei*, *Leuconostoc mesenteroides*, and *Streptococcus thermophilus* exhibit proteolytic activity, with proteolysis diameters ranging between 24 ± 5.30 mm and 27.5 ± 10.61 mm.

On the other hand, the findings regarding the antibacterial properties of the LAB isolates are in agreement with those of Tahlaiti et al. [31], who revealed that the lactic acid bacteria strains *Lb. plantarum* M6, R27 strain, *Levilactobacillus brevis* BL8 strain, and *Pediococcus acidilactici* M54 strain from Algerian fermented wheat (Hamoum) exhibit a strong effect on *E. coli*, *P. aeruginosa*, and *S. aureus*. This result could be attributed to the synthesis of antibacterial metabolites, particularly bacteriocins, organic acids, and hydrogen peroxide [39], as well as exopolysaccharides (EPSs) [40]. Also, it is observed that protein denaturation, related to inflammation, is influenced by lactic acid bacteria, whose protective capacity varies according to the strains and the bioactive molecules produced. Studies such as those by Khan et al. [40] and Jain and Mahta [41] have shown that strains such as *Ligilactobacillus agilis*, *Lb. casei*, and *Enterococcus faecium* inhibit the denaturation of bovine serum albumin, with significant anti-inflammatory effects.

At the same time, the results regarding the antioxidant activity reveal that the LAB isolates exhibit significant antioxidant activity. This is reflected by a strong ability to scavenge the DPPH radical, a power to reduce iron, an inhibition of lipid peroxidation, and a capacity to produce GSH. Indeed, these isolates exhibit a DPPH° scavenging capacity comparable to that reported by Riane et al. [42] for lactic acid bacteria strains isolated from fermented milk (72% to 83%). Our results are also similar to those of Duz et al. [43], who observed a maximum activity of 90.34 ± 0.40% for the *Lb. plantarum* IH14L strain. Previous studies have notably shown that the antioxidant activity of *Lacticaseibacillus rhamnosus*, *Lactobacillus helveticus*, *Latilactobacillus sakei*, and *Lb. plantarum* is linked to the production of EPSs on the cell surface [44]. EPSs could trap free radicals by releasing active hydrogen or by combining with them to form stable compounds, and they also possess iron-reducing capacity [45]. At the same time, the results of plasma lipid peroxidation inhibition are similar to those of Lin and Chang [46], who exhibited that *Bifidobacterium longum* and *Lactobacillus acidophilus* inhibit the plasma lipid peroxidation with rates ranging from 11 to 29%. Another study conducted by Zhang et al. [47] revealed that lactic acid bacteria exert antioxidant activity against lipid oxidation by inducing the expression of antioxidant genes using adaptive mechanisms, particularly by chelating transition metals, which promote the formation of free radicals and lipid peroxidation. EPSs are also involved in inhibiting the formation of MDA. According to the research conducted by Li et al. [46], the EPSs of *Lb. plantarum* LP6 strain have the ability to enhance antioxidant enzymatic activities, maintain cellular integrity, and inhibit the lipid oxidation of PC12 cells exposed to H_2_O_2_. Also, the inhibitory effect on MDA formation by lactic acid bacteria is related to particular constituents of each bacterium, such as GSH, antioxidant enzymes, vitamins, amino acids, etc., and the different associated redox reactions [48].

## 5. Conclusions

This research suggests that the lactic acid bacteria strains identified as *Weissella confusa* TFWS1, *Weissella confusa* TFWS2, and *Lactiplantibacillus plantarum* TFWS3 obtained from traditional Algerian fermented wheat exhibit considerable probiotic potential due to their technological and functional properties. This study entailed an examination of fermented wheat as an original and promising source of bacterial strains endowed with probiotic characteristics and notable biological activities, a sector largely unexplored until now. These strains could be exploited for therapeutic or nutraceutical purposes, potentially serving as a starter culture or being used in the creation of novel fermented foods with beneficial properties.

## Figures and Tables

**Table 1:** Morphological, biochemical, and physiological characteristics of bacterial isolates from traditional fermented wheat.

Tests	LB1	LB2	LB3
Gram	+ *	+	+
Cell shape	cocci	cocci	rod
Catalase	−	−	−
Fermentation Type	Heterofermentative	Heterofermentative	Homofermentative
Arginine dihydrolase	+	+	−
Growth at temperature			
10 °C	+	+	+
30 °C	+	+	+
45 °C	+	+	+
Growth in NaCl (%)			
6.5	+	+	+
9.5	−	−	−

* (+) Positive results. (−) Negative results.

**Table 2:** Survival rate of all strains expressed as percentage of viability under acidic pH and bile salts conditions at 37 °C.

Bacterial Strains	pH 3.0 (3 h)	Bile Salts 0.3% (4 h)
LB1	54.13± 0.001 ^a^	88.84 ± 1.03 ^b^
LB2	40.23 ± 0.03 ^b^	75.74 ± 0.71 ^c^
LB3	52.93 ± 0.003 ^a^	97.54 ± 0.65 ^a^

The results are expressed as means ± SE (*n* = 3); ^a–c^ letters indicate significantly different between strains (*p* < 0.05).

**Table 3:** Antibiotic susceptibility of selected strains.

Antibiotics	LB1	LB2	LB3
Chloramphenicol (30 µg)	I	S	S
Colistin (10 µg)	I	I	I
Metronidazole (6 µg)	R *	R	R
Streptomycin (10 µg)	R	R	R
Penicillin (10 µg)	S	I	R
Ceftazidime (10 µg)	R	R	R
Gentamicin (10 µg)	S	S	I

* R: Resistant; I: intermediate; S: sensitive.

**Table 4:** Auto-aggregation and co-aggregation rates of selected strains.

Strains	Auto-Aggregation %	Co-Aggregation %	
		*E. coli*	*S. aureus*
LB1	65.5 ± 0.2 ^b^	6.78 ± 0.16 ^b^	50.62± 0.36 ^b^
LB2	64.5 ± 0.76 ^b^	6.19 ± 0.6 ^b^	50.2 ± 0.55 ^b^
LB3	73.83 ± 0.33 ^a^	27.85 ± 0.41 ^a^	60 ± 1.3 ^a^

The results are expressed as means ± SE (*n* = 3); ^a,b^ letters indicate significantly different between different strains (*p* < 0.05).

**Table 5:** Diameters (mm) of proteolysis displayed by bacterial strains.

Strains	T30 °C	T37 °C
LB1	23.5 ± 1.42 ^a^	20± 0.44 ^b^
LB2	19.75 ± 0.85 ^a^	20 ± 0.54 ^b^
LB3	19.5 ± 0.22 ^a^	19.3 ± 0.2 ^b^

The results are expressed as means ± SE (*n* = 3); ^a,b^ letters indicate significantly different between strains (*p* < 0.05).

**Table 6:** Antioxidant activity of bacterial strains.

	LB1	LB2	LB3
FRAP (µmol/L)	171.36 ± 17.77 ^b^	146.97 ± 1.25 ^b^	311.99 ± 1.55 ^a^
DPPH (%)	84.45 ± 0.42 ^a^	86.75 ± 0.65 ^a^	80.58 ± 0.15 ^b^
GSH (µmol/mg)	1.02 ± 0.03 ^a^	1.08 ± 0.05 ^a^	1.12 ± 0.04 ^b^
Lipid peroxidation (%)	39.18 ± 0.002 ^a^	25.63 ±0.003 ^b^	36 ± 0.001 ^a^

The results are expressed as means ± SE (*n* = 3); ^a,b^ letters indicate significantly different between strains (*p* < 0.05).

## Data Availability

The original contributions presented in this study are included in the article. Further inquiries can be directed to the corresponding author.

## Abbreviations

The following abbreviations are used in this manuscript:

LAB	Lactic acid Bacteria
CFU	Colony-forming Unit
ADH	Arginine dihydrolase
FRAP	Ferric-reducing antioxidant power
DPPH	2,2-diphenyl-1-picrylhydrazyl
GSH	Reduced glutathione
MDA	Malondialdehyde
